# Spatial Owner-Dog Seroprevalence of *Leptospira* spp. Antibodies in Oceanic Islands and Costal Mainland of Southern Brazil

**DOI:** 10.3390/tropicalmed8040229

**Published:** 2023-04-18

**Authors:** Aaronson Ramathan Freitas, Ruana Renostro Delai, Louise Bach Kmetiuk, Raquel Cuba Gaspar, Evelyn Cristine da Silva, Rafaella Martini, Leandro Meneguelli Biondo, Rogério Giuffrida, Ivan Roque de Barros Filho, Vamilton Alvares Santarém, Helio Langoni, Cláudia Turra Pimpão, Alexander Welker Biondo

**Affiliations:** 1 Department of Veterinary Medicine, Federal University of Paraná State, Curitiba 80035-050, PR, Brazil; 2 Department of Animal Science, School of Life Sciences, Pontifical Catholic University of Paraná, Curitiba 80230-130, PR, Brazil; 3 Department of Animal Production and Preventive Veterinary Medicine, School of Veterinary Medicine and Animals Science, São Paulo State University, Botucatu 18618-681, SP, Brazil; 4 Institute of Biotechnology, São Paulo State University (UNESP), Tecomarias Avenue, Botucatu 18607-440, SP, Brazil; 5 Department of Physiology, Federal University of Paraná State, Curitiba 81530-000, PR, Brazil; 6 National Institute of the Atlantic Forest (INMA), Santa Teresa 29650-000, ES, Brazil; 7 Laboratory of Veterinary Parasitology, Veterinary Teaching Hospital, University of Western São Paulo, São Paulo 19001-970, SP, Brazil

**Keywords:** one health, leptospirosis, zoonosis, Brazil, seroepidemiology, domestic animals health, human health

## Abstract

Leptospirosis has been described as a disease neglected worldwide. Affecting humans and animals, the disease is often related to poor environmental conditions such as lack of sanitation and presence of synanthropic rodents. Despite being considered as a One Health issue, no study has focused on comparing owner–dog seroprevalence between islands and seashore mainland. Accordingly, the present study assessed anti-*Leptospira* spp. antibodies by applying microscopic agglutination test (MAT) methods to *Leptospira* and assessing associated risk factors via univariate and multivariate logistic regression analysis of owners and their dogs in islands and seashore mainland of southern Brazil. No anti-*Leptospira* spp. Seropositivity was found in 330 owner serum samples, while dogs presented an overall seroprevalence of 5.9%. All seropositive dogs reacted to serogroups of *Leptospira interrogans*, including 66.7% of Pyrogenes, 44.4% Canicola, 22.2% Icterohaemorrhagiae, 16.7% Australis; six reacted to more than one serogroup. No association was found among seropositivity and epidemiological variables, except that neighborhood dogs were less likely to be seropositive. Although no seropositivity was observed in owners, seropositivity in dogs had the potential to indicate such species as being sentinels for environmental exposure and potential human risk of infection.

## 1. Introduction

Leptospirosis, described as a tropical disease caused by bacteria of the genus *Leptospira*, is considered one of the most neglected zoonoses worldwide [1,2]. Of global public health importance, the disease has mammals as reservoirs and infection with it may occur due to contact between soil or water by contaminated *Leptospira* spp., shed from urine of mammalian hosts [3], with Norway (*Rattus norvegicus*) and black (*Rattus rattus*) rats, the most common primary reservoirs [3].

The disease transmission routes have been reportedly associated with socioenvironmental issues including inadequate infrastructure and sewage, garbage accumulation, and rodent proliferation, rendering vulnerable populations in tropical countries as more susceptible to disease [4,5,6,7]. Groups at risk may include gardeners, veterinarians, sewage workers, fishermen, and tourists visiting endemic regions [2].

Traditional communities living on islands in southern Brazil may be at aggravated risk due to the lack of public or private healthcare access for humans and dogs [8,9,10]. In such a scenario, a One Health approach may provide a comprehensive understanding of pathogen occurrence and dynamics according to social and economic characteristics of local populations. Accordingly, the aim of this study was to serologically assess owners and their dogs living on oceanic islands and mainland seashore areas for the presence of anti-*Leptospira* spp. antibodies and potential associated risk factors.

## 2. Materials and Methods

### 2.1. Study Design

This study was designed as a cross-sectional and epidemiological survey of owner– dog population living in oceanic islands and coastal mainland of State of Paraná, southern Brazil, and was conducted from July 2019 to February 2020. The search herein was approved by the Ethics Committee of Animal Use by the Federal University of Parana (protocol 036/2021) and the Ethics Committee in Human Health at the Brazilian Ministry of Health (protocol 46994521.0.0000.0102).

### 2.2. Local of Study

**Figure 1:**
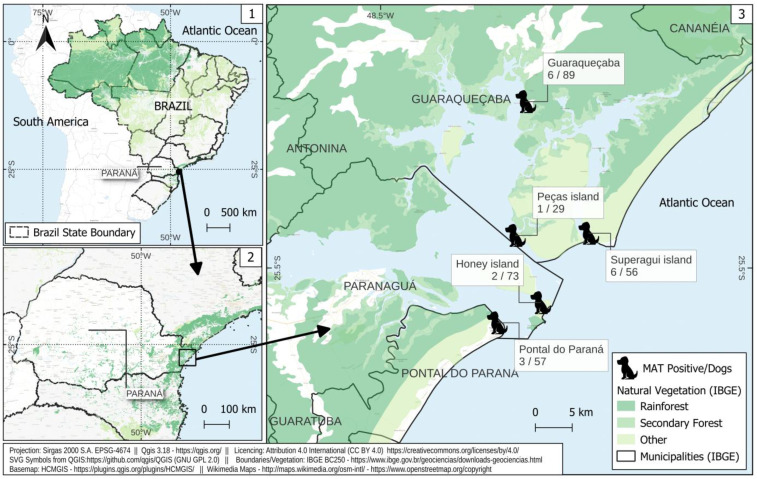
Sampling locations and frequency of anti-*Leptospira* spp. antibodies in owners and dogs from island and seashore mainland areas of southern Brazil. 1. Map of Brazil; 2. Map of Parana State; 3. Map islands and seashore mainland studied areas.

The study was performed in three oceanic islands, Ilha do Mel Island (25°32′32.4″ S and 48°18′15.1″ W), Superagui Island (25°27′27.1″ S and 48°14′43.6″ W) and Peças Island (25°27′36.5″ S and 48°20′06.5″ W), and two continental coastal mainland areas, the cities of Guaraqueçaba (25°17′47.9″ S and 48°19′20.8″ W) and Pontal do Paraná (25°34′01.2″ S and 48°21′26.6″ W), located in the Paraná State, southern Brazil. The islands studied herein were located within environmental protection units, such as the Ilha do Mel Island State Park and Superagui Island National Park, both of which are inserted in the largest unfragmented area of the Brazilian Atlantic forest biome (Figure 1) [11].

At the time, Ilha do Mel Island presented a local community of traditional culture with only 1100 permanent inhabitants, and a floating population that increased up to 5 times during the summer season. Distributed in five villages, tourism was the main source of income on Ilha do Mel Island [11]. The Superagui Island and Peças Islands have several villages with communities of traditional artisanal fishermen [12]. Finally, the closest two cities on the coastal mainland areas, Guaraqueçaba and Pontal do Paraná, were sampled for continental comparison [13].

### 2.3. Blood Samples

Human participants were individually sampled by arm cephalic puncture conducted by certified nurses and after signed consent had been obtained. Dogs were sampled by jugular puncture conducted by certified veterinarians and after signed owner consent had been obtained. Additionally, neighborhood dogs were sampled herein. Neighborhood dogs was defined as semi-restricted or free-range animals with undistinguished owners due to semi-dependence on one or more families for food and shelter [14]. All samples were then placed in serum-separating tubes, centrifuged at 800× *g* for five minutes, serum-separated and stored at −20 °C until processing.

A total of 333 serum samples were collected from island and seashore dog owners, with 3 serum samples insufficient for serological testing. Of the remaining 330 samples, 113 (34.2%) were from Ilha do Mel Island, 54 (16.4%) from Superagui Island, 27 (8.2%) from Peças Island, 45 (13.6%) from Pontal do Paraná, and 91 (27.6%) from Guaraqueçaba. A total of 304 serum samples were collected from dogs living on islands and the seashore mainland of Paraná state, including from 73 (24.0%) dogs of Ilha do Mel Island, 56 (18.4%) of Superagui Island, 29 (9.5%) of Peças Island, 57 (18.7%) of Pontal do Paraná, and 89 (29.3%) of Guaraqueçaba.

### 2.4. Epidemiological Data Collection

An epidemiologic questionnaire was applied for human risk factor analysis which measured potential exposure to *Leptospira* spp. and included owner information on age, sex, education level, household income and location, animal ownership (dog or cat), source of drinking water, and whether food was washed before eating. The risk factors attributed to dog analysis were also based on data obtained from epidemiological questionnaires and included the location of residence, sex, daily diet, source of drinking water, beach access, and hunting habits.

### 2.5. Serology Analysis

The microscopic agglutination test (MAT) was performed using a collection of 30 different serogroup of *Leptospira* spp. The serogroups included in the study were: Australis, Bratislava, Autumnalis, Castellonis, Bataviae, Canicola, Whitcombi, Cynopteri, Djasiman, Sentot, Grippotyphosa, Hebdomadis, Copenhageni, Icterohaemorraghiae, Javanica, Panama, Pomona, Pyrogenes, Hardjo, Wolffi, Shermani, Tarassovi, Andamana, Patoc, Prajtino, Minis, CTG, Bovis, and Guaricura e Nupezo-01. The bacterial suspensions used in the MAT were prepared from reference strains maintained at the Zoonosis Research Nucleus (NUPEZO) of the Faculty of Veterinary Medicine and Animal Science, São Paulo State University (UNESP)—Campus Botucatu. Strains were maintained in Ellinghausen–McCullough–Johnson–Harris (EMJH) media at 28 °C, with weekly evaluation and replication. A 1:100 dilution was used as the cutoff point for the serological test, as also established in previous studies [6,15,16]. All samples were analyzed at dilutions ranging from 1:100 to 1:3200, considering the highest positive dilution to possess the serum title [15,17,18]. For seroreactivity assessment, the sample was considered reactive when at least 50% of the leptospiral bacterial suspensions were found to be agglutinated in the test reading, with 100× magnification under a dark field microscope.

### 2.6. Statistical Analysis

Univariate analysis was conducted to assess the association between anti-*Leptospira* agglutinins and risk factors in dogs, using either a chi-square test or a Fisher exact test when necessary. Dogs with inconsistent or missing information were excluded from the univariate and multivariate analyses. Variables with a *p* value < 0.20 were included in a multivariate analysis using a logistic regression approach. Although the decision to only include variables with a *p* value < 0.20 may appear to be highly rigorous, the use of such an approach in a multivariate logistic regression analysis has been common practice in epidemiological research. In the initial bivariate analysis, the significance of a variable may be underestimated due to confounding from other variables. Thus, variables with a *p* value < 0.20 may be considered for inclusion in a multivariate analysis, both for controlling for potential confounding and determining whether the variable has been independently associated with the outcome of interest, as commonly and traditionally applied in multivariate logistic regression. To avoid problems related to nonconvergence due to small-sample bias in logistic regression, Firth’s penalized likelihood was applied to estimate the coefficients of the model [19]. The odds ratio (OR) was calculated with 95% confidence intervals (95% CI) for uni- and multivariate analysis. All statistical analysis was conducted in R software v. 4.2.2, with logistf package, at a 5% significance level [20,21].

## 3. Results

No anti-*Leptospira* agglutinins were detected in human serum samples using a cut-off point of 1:100 in MAT.

In overall, 18/304 (5.9%; 95% CI = 3.8–9.2%) dogs were seropositive with *Leptospira* spp. Seropositivity was detected in 2/73 (2.7%) dogs of Ilha do Mel Island, 6/56 (10.7%) of Superagui Island, 1/29 (3.5%) of Peças Island, 3/57 (5.3%) of Pontal do Paraná, and 6/89 (6.7%) of Guaraqueçaba.

All dogs were found to be seroreactive to the interrogans genospecies, with positivity observed for serogroups Pyrogenes (12/18 = 66.7%), Canicola (8/18 = 44.4%), Icterohaemorrhagiae (4/18 = 22.2%), and Australis (3/18 = 16.7%). Serological titers ranged from 1:100 to 1:800, with six dogs (6/18 = 33.3%) presenting reactivity to more than one serogroup (Supplementary Table S1).

The screening metho based on univariate analysis identified two predictors (*p* < 0.20) of multiple logistic regression: being a community dog (*p* = 0.017) and the ingestion of springer water (*p* = 0.085). Logistic regression model for the dogs provided a summary of the associations between variables and the presence of anti-*Leptospira* spp. antibodies (Table 1). Neighborhood dogs were considered to be less exposed to Leptospira spp., and consequently this was a protective factor for the presence of anti-*Leptospira* spp. antibodies (OR: 0.16; 95% CI: 0.04–0.60; *p* = 0.017). No association was verified between seropositivity and the other tested variables.

## 4. Discussion

To the authors’ knowledge, this is the first comparative study of *Leptospira* spp. exposure between dogs and their owners from mainland and seashore areas of Brazil.

Leptospirosis has been reportedly described as a disease affecting both humans and animals. It has a significant impact on public health, particularly in low-income countries [2]. The primary factors associated with leptospirosis include poor sanitation, climatic conditions, and overcrowding human populations [22]. Leptospirosis indicates as an example of a tropical neglected disease which requires a collaborative and interdisciplinary One Health approach (human medicine, veterinary medicine, and environmental health) involving experts from different areas. In addition, the study herein may be an important step to better understanding the seashore pattern of owner and dog leptospirosis.

Leptospirosis is considered to constitute a major public health threat in Paraná state, with a yearly average of 4.3 cases per 100,000 inhabitants [23]. Among the seven municipalities within the Paraná state coastline, the Brazilian Notifiable Diseases Information System (SINAN, Brazilian Ministry of Health), a publicly available online database maintained by the Data Information Department of the Unified Health System (DATASUS), recorded a total of 198 human leptospirosis cases, corresponding to 5.3 cases per 100,000 inhabitants [24].

The human population studied herein lived in subtropical areas with regular rainfall regime under precarious sanitary conditions, working in fishing and touristic activities, and having frequent soil contact [7]. Despite the favorable outdoors conditions, the absence of seropositivity in humans for Leptospira spp. in humans herein could be attributed to different factors. Firstly, the present study voluntarily recruited healthy participants, corroborating the results of surveys conducted in similar isolated human communities, such as Fernando de Noronha’s Oceanic Island [25] and the metropolitan areas of southern Brazil [26], with seropositivity of 1.17% and 1.84% for healthy owners, respectively. Secondly, the study confirmed the low likelihood of leptospirosis exposure when engaged in human occupational activities on environmentally protected islands, including forbidden livestock raising, slaughter, and wildlife hunting [27]. Such activities account for nearly 30% of human leptospirosis cases worldwide [28].

Although surveyed areas discussed herein present low overall seroprevalence, high prevalence rates of leptospirosis have been reported for dogs living in costal and island regions worldwide [29,30]. Nevertheless, the presence of seropositive dogs was observed in all surveyed areas, highlighting the importance of dogs as sentinels for leptospirosis contact, particularly in isolated regions [31]. In addition, dogs herein were also found to potentially play a pivotal role as domestic animal carriers of *Leptospira* spp., given the local livestock farming prohibition. The present study has also contributed to the One Heath approach to addressing owner–dog leptospirosis in isolated communities, with insights ranging from pet-only specifics to public health assessment and concern, supporting public policies on effective surveillance, prevention and control measures [32].

Herein, Pyrogenes was the most prevalent serogroup detected in dog samples. Conversely, Canicola and Icterohaemorragiae have been considered the most widely prevalent serogroups in the dogs of Brazil [16,33,34,35]. It was noteworthy that one tested dog presented a high serological titer, which was probably due to active infection. As in the present study, Pyrogenes has mostly been found in dogs previously [33], confirming that serogroup distribution and prevalence vary according to geographical location. Detection of Icterohaemorrageae serogroup in dogs, particularly in the Superagui Island, suggests their exposure to synanthropic urban rodents, mainly to *Rattus novergicus* and *Rattus rattus* [36]. In such a scenario, rodents may play a crucial role along with dogs in spreading the pathogen bacteria.

Although dog seropositivity to a specific serogroup can arise from natural infection, vaccination, or colostrum antibodies [37], dogs had no referred vaccination, lowering false-positive chances by vaccinal cross-reaction. Regardless, vaccination against leptospirosis should be encouraged in areas with high endemicity. The Canicola and Icterohaemorrhagiae serogroup have been present in vaccines in Brazil, while another emerging and non-vaccinal serogroup, such as Bratislava, Hardjo, Pomona, and Grippotyphosa [38,39,40] were not found in the present study.

The results of both univariate and logistic models revealed that the neighborhood dogs analyzed herein were less exposed to *Leptospira* spp. The underlying factors contributing to this finding remain unclear, especially since neighborhood dogs may be more exposed to outdoors conditions, thus increasing the *Leptospira* spp. infection risk, as previously shown in relation to homelessness, garbage scavenging, and puddle drinking [34]. In addition to the unlikelihood of vaccinal antibodies, the dogs evaluated herein seroreacted with non-common vaccinal serogroups. As potential protective behavior, neighborhood dogs may find sandy beaches as their resting places, these being areas with less rainwater and urine accumulation. As important, dogs may have limited exposure to commensal rodents, which are reportedly most prevalent in both indoor and household environments [26].

Despite the ability of *Leptospira* spirochetes to survive in seawater, rain or inland fresh water, particularly in high-humidity areas [34], factors of environment, sex, food source, water source, and access to different environments were not associated with dog seropositivity. Nonetheless, this study was an initial survey, and such risk factors should not be completely ruled out as potential exposure sources. The primary limitation of this study was that determinations of the intensity and frequency of risk factors relied solely on information given by the dog owner. Moreover, identifying the likely source of exposure by the application of a semi-structured questionnaire was difficult due to the complex and overlapping nature of risk activities.

Interestingly, the majority of owners reported dog hunting behavior as being significant, particularly towards rodents and other small animals. This was a factor which was not statistically associated with Leptospira seropositivity. As similar investigations carried out in Chile and in Brazil have also failed to identify hunting behavior as a risk factor for leptospirosis in dogs [41,42], the role of native and invasive rodents remains unclear.

The present investigation also had technical limitations as the applied antigen panel had the potential to fail to detect local prevalent serogroups, resulting in false-negative MAT results [43]. In addition, the cross-sectional study herein may have failed to detect any seasonal influence on *Leptospira* transmission. Finally, while serological techniques like MAT may be useful in detecting dog response, urine sampling and bacterial isolation should be always employed to genetically identify and characterize pathogenic *Leptospira* [44].

## 5. Conclusions

In the study herein, low *Leptospira* seropositivity was found in dogs from islands and the seashore mainland areas, with no presence detected in their dog owners. The unexpected protective factor of neighborhood dogs for presence of anti-*Leptospira* spp. antibodies may be related to the lower levels of exposure prevalent in non-household environments. Although no seropositivity was observed in owners, dog seropositivity may indicate the presence of such species and act as an early One Health sentinel for potential environmental exposure and human infection risk.

## Figures and Tables

**Table 1:** Associated risk factors for anti-*Leptospira* agglutinins in dog population of islands and the seashore mainland of Paraná state, southern Brazil (N = 304), by uni- and multivariate statistical analysis.

	MAT Test Result		Univariate Analysis		Multivariate Analysis	
	Positive (%)	Negative (%)	OR (CI 95%)	*p* Value	OR (CI 95%)	*p* Value
Variable	18 (5.9)	286 (94.1)				
Local				1.00		
Seashore	9 (50.0)	137 (47.9)	Reference [1.0]			
Islands	9 (50.0)	149 (52.1)	1.09 [0.41–2.90]			
Sex				0.236		
Female	6 (33.3)	145 (50.7)	Reference [1.0]			
Male	12 (66.7)	141 (49.3)	0.49 (0.16–1.32)			
Community dog				0.042 *		0.017 *
No	15 (83.3)	275 (96.2)	Reference [1.0]		Reference [1.0]	
Yes	3 (16.7)	11 (3.85)	0.20 (0.05–0.98)		0.16 (0.04–0.60)	
Ingestion of commercial dog food ^†^				0.584		
No	1 (5.56)	13 (4.56)	Reference [1.0]			
Yes	17 (94.4)	272 (95.4)	1.38 (0.05–7.71)			
Ingestion of homemade food ^†^				1.0		
No	8 (44.4)	125 (43.9)	Reference [1.0]			
Yes	10 (55.6)	160 (56.1)	1.03 (0.38–2.72)			
Ingestion of drinkable water				0.285		
No	9 (50.0)	99 (34.6)	Reference [1.0]			
Yes	9 (50.0)	187 (65.4)	1.88 (0.70–5.04)			
Ingestion of water from artesian well				1.0		
No	17 (94.4)	257 (89.9)	Reference [1.0]			
Yes	1 (5.56)	29 (10.1)	1.70 (0.33–41.9)			
Ingestion of springer water				0.085		0.102
No	9 (50.0)	206 (72.0)	Reference [1.0]		Reference [1.0]	
Yes	9 (50.0)	80 (28.0)	0.39 (0.15–1.05)		0.42 (0.15–1.17)	
Access to trails				0.409		
No	5 (27.8)	116 (40.6)	Reference [1.0]			
Yes	13 (72.2)	170 (59.4)	0.58 (0.18–1.59)			
Access to the beach ^†^				0.648		
No	6 (33.3)	119 (41.8)	Reference [1.0]			
Yes	12 (66.7)	166 (58.2)	0.71 (0.24–1.90)			
Access to the forest ^†^				0.792		
No	9 (50.0)	160 (56.1)	Reference [1.0]			
Yes	9 (50.0)	125 (43.9)	0.78 (0.29–2.09)			
Hunting rodents ^†^				0.504		
No	12 (75.0)	202 (82.1)	Reference [1.0]			
Yes	4 (25.0)	44 (17.9)	0.64 (0.21–2.45)			
Hunting other animals ^†^				1.0		
No	10 (62.5)	162 (65.1)	Reference [1.0]			
Yes	6 (37.5)	87 (34.9)	0.89 (0.31–2.74)			

* *p* < 0.05: †: variables with missing information; in columns reporting Odds Ratio estimates, “Reference group [1.0]” indicates the baseline group for comparison with the exposed group.

## Data Availability

Not applicable.

## Supplementary Materials

The following supporting information can be downloaded at: https://www.mdpi.com/article/10.3390/tropicalmed8040229/s1, Table S1: Serogroups and serum titles of dogs on islands and the seashore mainland of Paraná state, southern Brazil.
