# Supplementary material for: Spatial Owner-Dog Seroprevalence of Leptospira spp. Antibodies in Oceanic Islands and Costal Mainland of Southern Brazil
Source: Trop Med Infect Dis. 2023 Apr 18;8(4):229. doi: 10.3390/tropicalmed8040229 (PMC10141485; doi:10.3390/tropicalmed8040229)
Supplement: Supplementary file 1 [file tropicalmed-08-00229-s001.zip › tropicalmed-2311605-supplementary.pdf]

Supplementary Table 1 –Serogroups and serum titles of dogs on islands and the seashore mainland of Paraná state, southern Brazil

Local	Dog	Serogroup	Sorovar (Title)
Ilha do Mel Island	1	Pyrogenes	15 (100)
	2	Canicola	5 (100)
		Pyrogenes	15 (100)
Superagui Island	3	Pyrogenes	15 (200)
	4	Canicola	5 (100)
		Icterohaemorraghiae	11A (200)
		Pyrogenes	15 (100)
	5	Pyrogenes	15 (400)
	6	Canicola	5 (200)
		Pyrogenes	15 (800)
	7	Canicola	5 (100)
		Icterohaemorraghiae	11A (100)
		Pyrogenes	15 (200)
	8	Canicola	5 (100)
		Icterohaemorraghiae	11A (100)
		Pyrogenes	15 (200)
Peças Island	9	Canicola	5 (400)
Guaraqueçaba	10	Pyrogenes	15 (100)
	11	Australis	1B (100)
	12	Pyrogenes	15 (100)
	13	Pyrogenes	15 (100)
	14	Pyrogenes	15 (100)
	15	Canicola	5 (400)
Pontal do Paraná	16	Canicola	5 (100)
		Icterohaemorraghiae	11A (100)
	17	Australis	1A (100)
	18	Australis	1A (100)
